# Identification of Coenzyme Q10 and Skeletal Muscle Protein Biomarkers as Potential Factors to Assist in the Diagnosis of Sarcopenia

**DOI:** 10.3390/antiox11040725

**Published:** 2022-04-06

**Authors:** Chi-Hua Yen, Po-Sheng Chang, Yu-Hsun Chang, Ping-Ting Lin

**Affiliations:** 1 School of Medicine, Chung Shan Medical University, Taichung 402367, Taiwan; cshy352@csh.org.tw; 2 Department of Family and Community Medicine, Chung Shan Medical University Hospital, Taichung 402367, Taiwan; 3 Department of Nutrition, Chung Shan Medical University, Taichung 402367, Taiwan; s0746002@gm.csmu.edu.tw (P.-S.C.); s0945004@gm.csmu.edu.tw (Y.-H.C.); 4 Graduate Program in Nutrition, Department of Nutrition, Chung Shan Medical University, Taichung 402367, Taiwan; 5 Department of Nutrition, Chung Shan Medical University Hospital, Taichung 402367, Taiwan

**Keywords:** coenzyme Q10, sarcopenia, myokines, skeletal muscle protein biomarkers, diagnosis

## Abstract

The aim of this study was to explore the use of coenzyme Q10 and skeletal muscle protein biomarkers in the diagnosis of sarcopenia. Subjects with or without sarcopenia were recruited. The anthropometric, muscle strength and endurance measurements were assessed. Muscle proteins (albumin and creatine kinase), myokines (irisin and myostatin), and the coenzyme Q10 level were measured. Approximately half of the subjects suffered from a low coenzyme Q10 concentration (<0.5 μM). The levels of creatinine kinase and irisin were significantly lower in subjects with sarcopenia (*p* ≤ 0.05). In receiver operating characteristic analyses, irisin and creatine kinase showed a better prediction capability for sarcopenia (area under the curve, irisin: 0.64 vs. creatinine kinase: 0.61) than other biomarkers. Additionally, a low level of irisin (<118.0 ng/mL, odds ratio, 6.46, *p* < 0.01), creatine kinase (<69.5 U/L, odds ratio, 3.31, *p* = 0.04), or coenzyme Q10 (<0.67 μM, odds ratio, 9.79, *p* < 0.01) may increase the risk for sarcopenia even after adjusting for confounders. Since the levels of coenzyme Q10 and muscle biomarkers, such as irisin and creatine kinase, are associated with sarcopenia, we suggest they could be used as candidate markers to assist in the diagnosis of sarcopenia.

## 1. Introduction

Sarcopenia is an aging-related disease that may affect an individual’s quality of life and mortality [1]. Factors such as static status, malnutrition, hormonal changes, or inflammatory status may interfere with skeletal muscle synthesis to increase the risk of sarcopenia [2,3]. Taiwan has entered the stage of an aged society. Previous research has indicated that the prevalence of sarcopenia is 6.8% in the elderly, and the prevalence for men and women is 9.3% and 4.1%, respectively [4]. This means that sarcopenia has become an important issue for the health of older adults. The diagnosis of sarcopenia in Taiwan is determined according to a guideline from the Asian Working Group for Sarcopenia. If an individual has a low calf circumference, then their skeletal muscle strength and function can be further evaluated. Dual energy X-ray absorptiometry (DXA) or bioelectrical impedance analysis (BIA) can be used to assess the skeletal muscle mass index of the limbs to identify an individual with low skeletal muscle strength or function [5]. Compared to the BIA measurement, DXA can measure skeletal muscle mass more accurately because the measurement is not affected by changes in hydration, soft-tissue edema, exercise status, or food intake [6,7]. However, it is not easy to perform the DXA measurement on a large population [8]. Thus, it is worth exploring alternative measurements that could more easily assist in the diagnosis of sarcopenia in clinical practice.

Using biomarker measurements to assess the diagnosis of sarcopenia is a promising methodological strategy. A recent report proposed that myokines may be a biomarker of frailty status [9]. Myokines, including irisin and myostatin, participate in skeletal muscle protein synthesis. Irisin assists in utilizing glucose and stimulates fatty acid oxidation in skeletal muscle tissue [10]. Irisin has been found to be positively correlated with skeletal muscle mass and skeletal muscle strength in animal models [11]. However, myostatin negatively regulates skeletal muscle growth and may activate the ubiquitin–proteasome system in skeletal muscle tissue to induce skeletal muscle loss [12,13,14]. In addition, mitochondrial dysfunction has also been proposed to change skeletal muscle innervation, leading to weakness and sarcopenia [15]. Coenzyme Q10 is a mitochondrial nutrient that acts as a transmitter of electrons in the mitochondria and is response for energy production [16]. Studies have observed that elderly individuals may suffer from a low level of coenzyme Q10, and this low level may be correlated with the incidence of sarcopenia [17,18,19,20]. Serum albumin [21,22] and creatine kinase [23] are associated with the skeletal muscle mass status, and they are commonly used as clinical indicators for assessing the protein status of patients. Thus, the aim of this study was to explore these hematological biomarkers to determine the status in assisting in the diagnosis of sarcopenia.

## 2. Materials and Methods

### 2.1. Subjects and Study Design

The present study was a cross-sectional study. We recruited subjects with sarcopenia and without sarcopenia. The included participants were ≥ 40 years old. The diagnosis of sarcopenia was defined according to the Asian Working Group for Sarcopenia [5], which was assessed by appendicular skeletal muscle mass index and skeletal muscle strength and endurance. The exclusion criteria for both the sarcopenia and non-sarcopenia groups were as follows: (1) subjects who were diagnosed with cancer, severe heart, lung, liver, and kidney disease; (2) the consumption of coenzyme Q10 supplement; (3) the use of anti-hyperlipidemia or anti-thrombin agents in the past month; and (4) knee replacement surgery. This study was approved by the Institutional Review Board of Chung Shan Medical University Hospital, Taiwan (CSMUH No: CS2-20137). Each subject provided written informed consent to participate in the study.

### 2.2. Data Collection, Anthropometry, and Skeletal Muscle Function Measurements

The characteristics of the subjects, such as age, gender, and life habits, were collected from a questionnaire. Height and weight were measured, and body mass index was calculated. Blood pressure was measured by a digital electronic sphygmomanometer (Hartmann Tensoval^®^ duo control, Heidenheim, Germany). A measuring tape was used to measure the waist, hip, and calf circumference. Skeletal muscle mass, including the whole skeletal muscle mass index, appendicular skeletal muscle mass index, and body fat, was measured by DXA (Hologic, ASY-05119, Marlborough, MA, USA). For the skeletal muscle strength and endurance measurements, the upper and lower limb skeletal muscle strength was evaluated as the handgrip and leg-back strength. Handgrip was assessed by a handgrip dynamometer (TAKEI, TKK-5401, Niigata, Japan). Leg-back strength was assessed by a back dynamometer (TAKEI, TKK-5402, Niigata, Japan). Upper limb endurance was assessed by dumbbell curls of the dominant hand. Subjects were instructed to flex and extend the elbow to lift either an 8 lb dumbbell for males or a 5 lb dumbbell for females for 30 s. Lower limb endurance was assessed by the chair-stand test. We recorded the time in seconds in which the subjects could stand from a chair and then return to sit in it 5 times in a row. Gait speed was assessed by the 6 min walk test. Subjects were asked to walk on a flat road for 6 min, and then the distance that the subjects walked was recorded. Furthermore, we used the short physical performance battery (SPPB) to assess the physical performance of these subjects. The SPPB includes a balance test, gait speed test, and chair-stand test [5,24]. The Strength, assistance in walking, rise from a chair, climb stairs, and falls (SARC-F) test was used to assess the risk of sarcopenia, which was indicated by a score higher than 4 points [25].

### 2.3. Biochemical Analysis

Fasting venous blood specimens were collected in vacutainers with K2-EDTA anticoagulant (Becton Dickinson, Franklin Lakes, NJ, USA) or without anticoagulant. Plasma and serum samples were prepared after centrifugation at 4 °C and 3000 rpm for 15 min. The biochemical data, including albumin, creatinine kinase, glucose parameters, and the lipid profile were measured by an automated chemistry analyzer (Beckman Coulter, DxC 800, Brea, California; Hitachi 7600-110, Tokyo, Japan).

### 2.4. Skeletal Muscle Protein Biomarkers Measurements

Serum irisin and myostatin levels were determined by enzyme-linked immunosorbent assay (ELISA) using commercially available human ELISA kits (CSB-EQ027943HU and CSB-E11300h, CUSABIO Technology, Houston, TX, USA).

### 2.5. Coenzyme Q10 Measurement

We used the high-performance liquid chromatography (HPLC) with an ultraviolet detector to measure the level of coenzyme Q10 [26]. The protein in the plasma was precipitated by propanol after centrifugation, and methanol was added to the supernatant at the same ratio. The liquid was filtered after mixing for HPLC analysis. Mixed methanol and ethanol were used as the mobile phase. The analysis column was a LiChroCART^®^RP-18 (Merck, Darmstadt, Germany), and the wavelength of the ultraviolet detector was set at 275 nm. The mean of intra-assay (*n* = 5) and inter-assay (*n* = 9) coefficients variability of coenzyme Q10 were 3.9% and 4.4%, respectively. The mean analytical recovery of coenzyme Q10 was 100.0%.

### 2.6. Statistical Analyses

All statistical analyses were performed using SigmaPlot software (Version 12.0, San Jose, CA, USA). The normality of each distribution was analyzed by the Shapiro–Wilk test. Student’s t test or the Mann–Whitney U test was used to compare the continuous variables between sarcopenia and non-sarcopenia subjects. The differences in categorical variables were examined by using the Chi-square test or Fisher’s exact test. The correlations between coenzyme Q10 and the skeletal muscle protein biomarkers and sarcopenia compositions were examined by Pearson’s correlation or Spearman’s rank correlation analysis. Receiver operating characteristic (ROC) analysis was used to identify the optimal cutoff value for coenzyme Q10 or the skeletal muscle protein biomarkers in predicting sarcopenia. The associations between the risk of sarcopenia and coenzyme Q10 and the skeletal muscle protein biomarkers were examined by logistic regression analyses. The statistical significance level was set at a *p* value ≤ 0.05.

## 3. Results

### 3.1. Characteristics of Subjects

Ninety-nine subjects were enrolled in the present study. Of these, 46 subjects had sarcopenia and 53 did not have sarcopenia. Table 1 shows the characteristic data of the subjects. The proportion of males was significantly higher in subjects with sarcopenia (*p* < 0.01). With regard to the anthropometric measurements, subjects with sarcopenia had significantly lower values for body mass index (*p* < 0.01), and both males and females in the sarcopenia group had significantly lower values for waist and calf circumference than those in the non-sarcopenia group (*p* < 0.05). Regarding lifestyle factors, the proportion of tobacco use was significantly higher in the subjects with sarcopenia than those without sarcopenia (*p* < 0.01).

### 3.2. Skeletal Muscle Protein Biomarkers and Skeletal Muscle Function

With regard to the skeletal muscle protein biomarkers, subjects with sarcopenia had significantly lower levels of creatine kinase (*p* = 0.05) and irisin (*p* = 0.02) than subjects without sarcopenia (Table 1). There was no significant difference in the levels of myostatin and coenzyme Q10 between the two groups (Table 1). Regarding skeletal muscle function, it is not surprising that subjects with sarcopenia had significantly lower skeletal muscle function parameter performance than those without sarcopenia (Table 1, *p* < 0.05).

### 3.3. Correlations between Coenzyme Q10 and the Skeletal Muscle Protein Biomarkers and Skeletal Muscle Functions

Table 2 shows the correlations between coenzyme Q10 and the skeletal muscle protein biomarkers and skeletal muscle functions. The coenzyme Q10 level was significantly positively correlated with the SPPB score (*p* = 0.04), and negatively correlated with the SARC-F score (*p* = 0.01). The level of serum albumin was significantly positively correlated with handgrip strength (in females), gait speed, and SPPB score (*p* < 0.05) and significantly negatively correlated with SARC-F score (*p* = 0.01). Creatine kinase activity was significantly positively correlated with whole and appendicular skeletal muscle mass index (*p* < 0.05). In addition, the level of irisin was significantly positively correlated with whole skeletal muscle mass index (in females), repetitions of dumbbell curls, and gait speed (*p* ≤ 0.05) in these subjects.

### 3.4. The ROC Curve Analysis of Coenzyme Q10 and the Skeletal Muscle Protein Biomarkers for Predicting Sarcopenia

**Figure 1:**
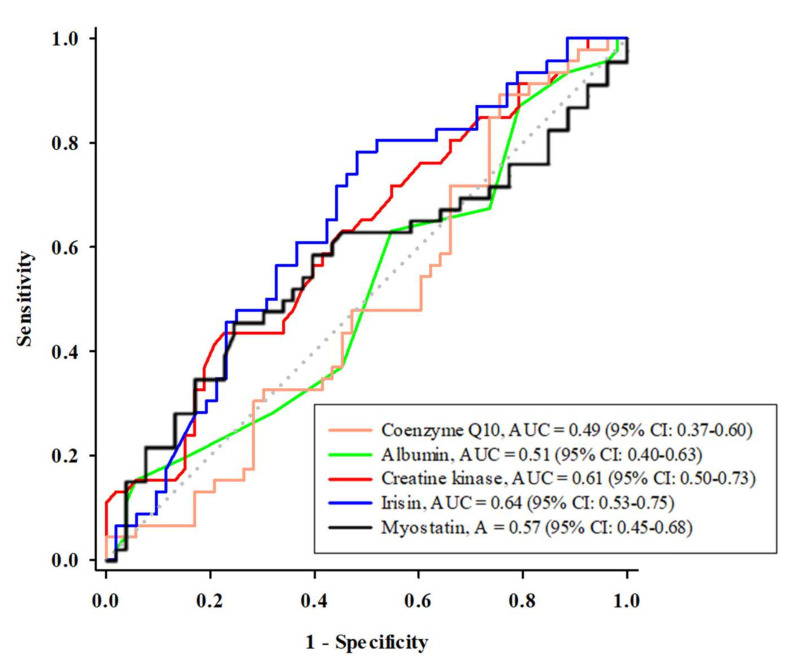
The receiver operating characteristic curve analysis of coenzyme Q10 and the skeletal muscle protein biomarkers in predicting sarcopenia. AUC, area under the curve; CI: confidence interval.

An optimal cutoff value for coenzyme Q10 or the skeletal muscle protein biomarkers was determined for their use in predicting sarcopenia by using ROC curves and is shown in Figure 1. The areas under the curves (AUCs) for irisin, creatine kinase, myostatin, albumin, and coenzyme Q10 were 0.64, 0.61, 0.57, 0.51, and 0.49, respectively. The optimal cutoff value for predicting sarcopenia was 118.0 ng/mL for irisin (sensitivity: 0.8; specificity: 0.5), 69.5 U/L for creatine kinase (sensitivity: 0.4; specificity: 0.8), 11.1 ng/mL for myostatin (sensitivity: 0.5; specificity: 0.8), 40.0 g/L for albumin (sensitivity: 0.2; specificity: 0.9), and 0.67 µM for coenzyme Q10 (sensitivity: 0.9; specificity: 0.2).

### 3.5. Associations between Sarcopenia and Coenzyme Q10 and the Skeletal Muscle Protein Biomarkers

We further examined the association between the risk of sarcopenia and the optimal cutoff values of coenzyme Q10 and the skeletal muscle protein biomarkers by using logistic regression analyses (Table 3). With the exception of albumin, the skeletal muscle protein biomarkers were significantly associated with the risk of sarcopenia (Models 1–3). Moreover, in Model 4, a lower level of coenzyme Q10 was significantly associated with an increased risk of sarcopenia (odds ratio: 3.70, *p* = 0.04), and after additionally adjusting for confounders (age, males, tobacco use, alcohol use, and physical activity), the level of irisin, creatine kinase, and coenzyme Q10 was significantly associated with an increased risk of sarcopenia (Model 5, irisin, odds ratio: 6.46, *p* < 0.01; creatine kinase, odds ratio: 3.31, *p* = 0.04; coenzyme Q10, odds ratio: 9.79, *p* = 0.01).

## 4. Discussion

In the present study, we tried to determine if coenzyme Q10 and the skeletal muscle protein biomarkers such as albumin, creatine kinase, irisin, and myostatin, could be used to assist in the diagnosis of sarcopenia. Among these candidates, we found that irisin, creatine kinase, and coenzyme Q10 may be predictive biomarkers for sarcopenia. Sarcopenia is often accompanied by the progression of aging. Although there was no significant difference in the level of coenzyme Q10 between the two groups (Table 1), the median level of the two groups was below the normal reference range (normal reference range: 0.5–1.7 µM) [27]. Approximately half of the subjects in this study suffered from a low coenzyme Q10 concentration. Age could be a factor that causes the low concentration of coenzyme Q10 [20]. Coenzyme Q10 is a nutrient that participates in the process of energy production in mitochondria [15,16]. Coenzyme Q10 plays an important energy source that could help to increase glycogen synthesis and reserves in skeletal muscle [28]. In the present study, we found that coenzyme Q10 was significantly correlated with SPPB and SARC-F scores (Table 2), implying that coenzyme Q10 affects muscular endurance. This finding is similar to our previous study showing that coenzyme Q10 was significantly correlated with skeletal muscle function in patients with osteoarthritis, and it may be related to its antioxidant capacity [29]. Recently, an observational study also found similar results; the researchers indicated that elderly individuals should maintain coenzyme Q10 level to avoid the risk of sarcopenia and frailty [30]. In addition, we noted that a low coenzyme Q10 status may be associated with an increased risk of sarcopenia; even after adjusting for confounders, statistical significance still existed (Table 3). Although the AUC value for coenzyme Q10 for predicting sarcopenia was not the highest, coenzyme Q10 may be depleted in subjects who suffer from aging. As a result, we suggest that targeting antiaging antioxidants, such as coenzyme Q10, could be considered a strategy to correct the low level of coenzyme Q10 in aging. Further interventional studies should be conducted to understand the effect of coenzyme Q10 supplementation on improving the status of coenzyme Q10 and the risk of sarcopenia.

Myokines, such as irisin and myostatin, are involved in skeletal muscle protein synthesis [9]. Irisin is released by skeletal muscle cells that express the transcription factor peroxisome proliferator-activated receptor-γ coactivator-1α (PGC-1α), which participates in pathways related to energy metabolism. In contrast to irisin, myostatin inhibits the positive modulation system of protein synthesis that is mediated by mammalian target of rapamycin (mTOR). Myostatin plays a role in skeletal muscle protein wasting by increasing degradation, as occurs in aging [31]. In the present study, we found a positive correlation between irisin and skeletal muscle mass and skeletal muscle strength (Table 3). Irisin is secreted through skeletal muscle contraction and acts as a skeletal muscle hormone to regulate skeletal muscle protein synthesis [9]. Among these predictors of sarcopenia, irisin had the highest AUC value and was the most consistently expressed marker in the statistical strength data compared with other predictors. The optimal cutoff point of irisin for the prediction of sarcopenia was 118 ng/mL (Figure 1). We further examined this level of irisin and the risk of sarcopenia by using logistic regression analyses. We found that a low level of irisin (<118 ng/mL) significantly increased the risk of sarcopenia (Table 3, Models 1–4), even after additionally adjusting for confounders (Table 3, Model 5). Another myokine, myostatin, negatively regulates skeletal muscle synthesis. In our participants, we found a slightly higher level of myostatin in subjects with sarcopenia than in those without sarcopenia, although the difference did not reach statistical significance (Table 1). Then, we examined the utility of myostatin in the diagnosis of sarcopenia, and the data showed that the AUC value of myostatin was the third highest (second to irisin and creatine kinase). However, the ability of myokines to predict sarcopenia was decreased after additional adjustment for confounders. Thus, we propose that irisin is a sensitive biochemical indicator of skeletal muscle weakness and has the potential to predict sarcopenia [32].

In addition to myokines, we successfully determined that the level of creatine kinase is also a good predictor for the diagnosis of sarcopenia, second only to irisin (Figure 1). The optimal cutoff value of creatine kinase was 69.5 U/L. The statistical significance of creatine kinase was as stable as that of irisin. A low level of creatine kinase may increase the risk of sarcopenia, even after adjusting for potential confounders (Table 3). The physiological function of creatine kinase is to convert creatine phosphate into creatine to generate energy, adenosine triphosphate (ATP), which helps skeletal muscle contraction [33]. Creatine kinase activity was significantly positively correlated with skeletal muscle mass, which is reduced due to aging, disease, or static state [34]. In the present study, creatine kinase was positively correlated with skeletal muscle mass (Table 2). Recently, some researchers have indicated that creatine kinase could predict mortality in subjects with chronic kidney disease [35] or predict sarcopenia in subjects with osteoarthritis [36]. Creatine kinase may reflect the individual’s skeletal muscle state [23], and we suggest that the level of creatine kinase could be considered an indicator for sarcopenia diagnosis in clinical application.

Serum albumin is often used as an indicator of protein status in a clinical setting. A low level of albumin implies the degradation of skeletal muscle protein, which also means that a person is at risk of malnutrition [37]. In the present study, we failed to detect a significant difference in the level of serum albumin between the sarcopenic and non-sarcopenic groups (Table 1). Although serum albumin correlated with gait speed and SPPB scores (Table 2), the AUC value of serum albumin was similar to coenzyme Q10. Furthermore, the optimal cutoff value of serum albumin was not associated with the risk of sarcopenia after adjusting for confounders (Table 3, Models 3–5). A report from a larger multi-medical center survey found that a marked decrease in albumin was related to skeletal muscle power in community-dwelling men [38]. Serum albumin is associated with skeletal muscle [21,22], and sarcopenia and low albumin may synergistically increase the risk of incident disability in older adults [39]. The subjects in our study did not appear to have low levels of albumin, and the mean serum albumin in both sarcopenic and non-sarcopenic groups was 44.0 g/L (Table 1). Therefore, we did not detect a relationship between serum albumin and sarcopenia. However, we still recommend that it is necessary to monitor the albumin status as aging progresses and sarcopenia progresses.

This study had certain limitations that should be mentioned. First, the sample size was small and the survey was only conducted in the Taiwanese population. Due to the small sample size in this study, the cutoff values for these biomarkers may only apply to these observational data. Thus, further large-scale studies are needed to confirm the results and provide sufficient information for clinical application. Second, this was a cross-sectional study, and we were unable to elucidate the causal relationship between coenzyme Q10 and the skeletal muscle protein biomarkers and sarcopenia. Third, diet may be a factor affecting the level of coenzyme Q10. However, the average dietary intake of coenzyme Q10 is only 3–6 mg [40]. Plasma coenzyme Q10 level may reflect dietary status rather than tissue level. Measuring skeletal muscle or tissue coenzyme Q10 may more accurately reflect a target tissue; however, it is not easy to perform in a clinical setting.

## 5. Conclusions

Subjects with sarcopenia had significantly lower levels of skeletal muscle protein biomarkers, such as creatine kinase and irisin, than those without sarcopenia. Notably, most subjects suffered from coenzyme Q10 deficiency. Since the levels of coenzyme Q10 and skeletal muscle protein biomarkers were significantly associated with the risk of sarcopenia, we suggest that coenzyme Q10, irisin, and creatine kinase may be candidate markers that could assist in the diagnosis of sarcopenia.

## Figures and Tables

**Table 1:** Characteristic data of subjects.

	Sarcopenia (*n* = 46)	Non-Sarcopenia (*n* = 53)	*p* Value
Age (years)	74.8 ± 7.4 (76.5)	72.0 ± 8.4 (72.0)	0.08
Males (n, %)	19 (41.3%)	7 (13.2%)	0.003
Body mass index (kg/m^2^)	19.8 ± 2.4 (20.1)	23.1 ± 2.9 (22.5)	<0.001
Systolic pressure (mmHg)	130.8 ± 19.5 (127.5)	134.8 ± 19.2 (134.0)	0.31
Diastolic pressure (mmHg)	76.5 ± 13.9 (75.5)	76.0 ± 10.6 (76.0)	0.98
Waist (cm)			
Males	81.5 ± 7.8 (83.5)	89.6 ± 7.4 (88.5)	0.03
Females	76.5 ± 6.9 (76.5)	84.9 ± 9.5 (84.8)	<0.001
Waist–hip ratio	0.88 ± 0.07 (0.89)	0.91 ± 0.08 (0.92)	0.07
Calf circumference (cm)			
Males	31.0 ± 2.2 (31.0)	34.7 ± 1.9 (35.0)	<0.001
Females	29.9 ± 2.6 (29.8)	32.3 ± 2.5 (31.9)	<0.001
SARC-F (points)	2.0 ± 2.3 (2.0)	2.0 ± 2.3 (1.0)	0.94
Tobacco use (n, %) ^1^	11 (23.9%)	0 (0.0%)	<0.001
Alcohol use (n, %) ^2^	5 (10.9%)	1 (1.9%)	0.09
Physical activity (n, %) ^3^	27 (58.7%)	32 (60.4%)	0.97
Serum albumin (g/L)	43.0 ± 4.0 (44.0)	44.0 ± 4.0 (44.0)	0.86
Fasting glucose (mmol/L)	6.4 ± 1.4 (5.8)	6.2 ± 1.2 (5.9)	0.95
Total cholesterol (mmol/L)	5.1 ± 1.3 (5.2)	5.4 ± 1.2 (5.6)	0.24
HDL-C (mmol/L)			
Males	1.2 ± 0.4 (1.2)	1.0 ± 0.2 (1.1)	0.26
Females	1.6 ± 0.4 (1.6)	1.6 ± 0.4 (1.6)	0.84
LDL-C (mmol/L)	2.9 ± 1.0 (2.9)	3.0 ± 0.8 (3.0)	0.47
Triglycerides (mmol/L)	1.1 ± 0.5 (0.9)	1.1 ± 0.6 (0.8)	0.63
Creatine kinase (U/L)	85.5 ± 40.8 (84.0)	105.7 ± 49.0 (100.0)	0.05
Irisin (ng/mL)	90.5 ± 105.3 (61.4)	159.7 ± 146.2 (123.0)	0.02
Myostatin (ng/mL)	9.5 ± 4.7 (10.0)	8.5 ± 3.9 (8.4)	0.26
Coenzyme Q10 (µM)	0.49 ± 0.16 (0.51)	0.50 ± 0.19 (0.48)	0.82
Muscle function			
WSMI (kg/m^2^)			
Males	14.8 ± 1.2 (14.8)	16.9 ± 1.3 (16.5)	<0.001
Females	12.5 ± 1.0 (12.7)	15.0 ± 1.3 (14.9)	<0.001
ASMI (kg/m^2^)			
Males	6.1 ± 0.6 (6.4)	7.5 ± 0.5 (7.2)	<0.001
Females	4.9 ± 0.3 (4.9)	6.1 ± 0.5 (6.0)	<0.001
Body fat (%)			
Males	21.4 ± 5.6 (22.0)	22.2 ± 3.3 (23.1)	0.74
Females	29.0 ± 4.8 (29.1)	29.3 ± 5.9 (29.2)	0.83
Handgrip strength (kg)			
Males	24.9 ± 5.1 (26.5)	27.0 ± 7.2 (25.7)	0.42
Females	15.6 ± 4.9 (16.8)	18.1 ± 4.6 (18.6)	0.03
Dumbbell curls (reps)	14.9 ± 5.6 (16.0)	19.6 ± 6.1 (19.0)	<0.001
Leg-back strength (kg)	34.4 ± 15.2 (29.5)	36.2 ± 14.4 (32.5)	0.33
Chair-stand test (s)	14.2 ± 4.7 (13.0)	12.6 ± 5.3 (11.6)	0.06
Gait speed (m/s)	0.70 ± 0.24 (0.67)	0.85 ± 0.29 (0.85)	0.01
SPPB (points)	9.3 ± 2.7 (10.0)	9.8 ± 2.8 (11.0)	0.13

Values are means ± SD (medians). ^1^ Tobacco use was defined as regularly smoking one or more cigarette per day or quitting ≥ 6 months. ^2^ Alcohol use was defined as regularly consuming one or more drink per day or quitting ≥ 6 months. ^3^ Subjects do exercise regularly at least 3 times per week. HDL-C, high-density lipoprotein cholesterol; LDL-C, low-density lipoprotein cholesterol; SARC-F, strength, assistance with walking, rise from a chair, climb stairs, and falls.

**Table 2:** Correlations between coenzyme Q10 and the skeletal muscle protein biomarkers and muscle functions.

	Coenzyme Q10 (μM)	Serum Albumin (g/L)	Creatine Kinase (U/L)	Irisin (ng/mL)	Myostatin (ng/mL)
	*r* ^1^ (*p* Value)				
WSMI (kg/m^2^)					
Total	−0.13 (0.21)	−0.04 (0.67)	0.20 (<0.05)	0.10 (0.34)	−0.02 (0.86)
Males	−0.06 (0.75)	0.22 (0.27)	0.20 (0.33)	0.22 (0.29)	−0.01 (0.96)
Females	−0.11 (0.38)	−0.10 (0.42)	0.18 (0.12)	0.30 (0.01)	−0.04 (0.74)
ASMI (kg/m^2^)					
Total	−0.09 (0.38)	0.01 (0.91)	0.31 (<0.01)	0.00 (0.97)	−0.01 (0.91)
Males	−0.06 (0.78)	0.21 (0.30)	0.34 (0.09)	0.16 (0.44)	0.03 (0.88)
Females	−0.03 (0.78)	−0.06 (0.62)	0.31 (<0.01)	0.22 (0.07)	−0.06 (0.60)
Handgrip strength (kg)					
Males	0.28 (0.17)	0.08 (0.70)	0.22 (0.28)	−0.25 (0.23)	0.06 (0.77)
Females	−0.03 (0.82)	0.38 (<0.01)	0.15 (0.20)	0.10 (0.39)	0.12 (0.32)
Dumbbell curls (reps)	0.09 (0.39)	0.12 (0.22)	0.00 (0.96)	0.20 (0.05)	0.03 (0.76)
Leg-back strength (kg)	0.00 (0.99)	0.10 (0.35)	0.11 (0.29)	−0.18 (0.08)	0.04 (0.67)
Chair-stand test (s)	−0.17 (0.10)	−0.20 (0.06)	0.06 (0.57)	−0.13 (0.21)	−0.03 (0.78)
Gait speed (m/s)	0.12 (0.26)	0.24 (0.02)	0.09 (0.38)	0.30 (<0.01)	0.13 (0.20)
SPPB (points)	0.21 (0.04)	0.29 (<0.01)	0.03 (0.75)	0.13 (0.19)	0.11 (0.26)
SARC-F (points)	−0.27 (0.01)	−0.25 (0.01)	−0.16 (0.10)	0.13 (0.19)	−0.07 (0.48)

^1^ Correlation coefficient. ASMI, appendicular skeletal muscle mass index; SARC-F, strength, assistance with walking, rise from a chair, climb stairs, and falls; SPPB, short physical performance battery; WSMI, whole skeletal muscle mass index.

**Table 3:** Associations between the risk of sarcopenia and coenzyme Q10 and the skeletal muscle protein biomarkers.

	Sarcopenia	
	Odds Ratio (95% Confidence Interval)	*p* Value
Model 1		
Irisin <118.0 ng/mL	4.85 (1.86–12.67) ^1^	<0.01
Creatine kinase <69.5 U/L	3.43 (1.30–9.05) ^2^	0.04
Model 2		
Irisin <118.0 ng/mL	5.26 (1.94–14.27) ^1^	<0.01
Creatine kinase <69.5 U/L	3.15 (1.18–8.41) ^2^	0.02
Myostatin >11.1 ng/mL	2.60 (1.02–6.64) ^3^	<0.05
Model 3		
Irisin <118.0 ng/mL	5.22 (1.85–14.75) ^1^	<0.01
Creatine kinase <69.5 U/L	3.13 (1.15–8.53) ^2^	0.03
Myostatin >11.1 ng/mL	2.60 (1.02–6.65) ^3^	<0.05
Albumin <40.0 g/L	1.04 (0.22–5.03) ^4^	0.96
Model 4		
Irisin <118.0 ng/mL	6.56 (2.23–19.30) ^1^	<0.01
Creatine kinase <69.5 U/L	2.65 (0.95–7.45) ^2^	0.06
Myostatin >11.1 ng/mL	2.58 (0.98–6.78) ^3^	0.05
Albumin <40.0 g/L	1.09 (0.21–5.71) ^4^	0.92
Coenzyme Q10 <0.67 µM	3.70 (1.06–12.88) ^5^	0.04
Model 5 (adjusted confounders ^6^)		
Irisin <118.0 ng/mL	6.46 (1.86–22.38) ^1^	<0.01
Creatine kinase <69.5 U/L	3.31 (1.09–10.10) ^2^	0.04
Myostatin >11.1 ng/mL	2.48 (0.83–7.40) ^3^	0.10
Albumin <40.0 g/L	0.80 (0.12–5.27) ^4^	0.82
Coenzyme Q10 <0.67 µM	9.79 (1.69–56.58) ^5^	0.01

^1^ Reference is as irisin ≥ 118.0 ng/mL. ^2^ Reference is as creatine kinase ≥ 69.5 U/L. ^3^ Reference is as myostatin ≤ 11.1 ng/mL. ^4^ Reference is as albumin ≥ 40.0 g/L. ^5^ Reference is as coenzyme Q10 ≥ 0.67 µM. ^6^ Confounders included age, males, tobacco use, alcohol use, and physical activity.

## Data Availability

The datasets generated and/or analyzed during the current study are available from the corresponding author on reasonable request.

## Abbreviations

ATP: adenosine triphosphate; AUC, area under the curve; BIA, bioelectrical impedance analysis; DXA, dual energy X-ray absorptiometry; ELISA, enzyme-linked immunosorbent assay; HPLC, high-performance liquid chromatography; mTOR, mammalian target of rapamycin; PGC-1α, peroxisome proliferator-activated receptor-γ co-activator-1α; ROC, receiver operating characteristic; SARC-F, strength, assistance in walking, rise from a chair, climb stairs, and falls; SPPB, short physical performance battery.

## References

[1] Dodds R.M., Sayer A.A. (2016). Sarcopenia; frailty and mortality: The evidence is growing. Age Ageing.

[2] Keller K., Engelhardt M. (2014). Strength and muscle mass loss with aging process. Age and strength loss. Muscles Ligaments Tendons J..

[3] Marzetti E., Calvani R., Tosato M., Cesari M., Di Bari M., Cherubini A., Collamati A., D’Angelo E., Pahor M., Bernabei R. (2017). SPRINTT Consortium. Sarcopenia: An overview. Aging Clin. Exp. Res..

[4] Kuo Y.H., Wang T.F., Liu L.K., Lee W.J., Peng L.N., Chen L.K. (2019). Epidemiology of Sarcopenia and Factors Associated with It Among Community-Dwelling Older Adults in Taiwan. Am. J. Med. Sci..

[5] Chen L.K., Woo J., Assantachai P., Auyeung T.W., Chou M.Y., Iijima K., Jang H.C., Kang L., Kim M., Kim S. (2020). Asian Working Group for Sarcopenia: 2019 Consensus Update on Sarcopenia Diagnosis and Treatment. J. Am. Med. Dir. Assoc..

[6] Chianca V., Albano D., Messina C., Gitto S., Ruffo G., Guarino S., Del Grande F., Sconfienza L.M. (2021). Sarcopenia: Imaging assessment and clinical application. Abdom. Radiol..

[7] Guglielmi G., Ponti F., Agostini M., Amadori M., Battista G., Bazzocchi A. (2016). The role of DXA in sarcopenia. Aging Clin. Exp. Res..

[8] Ceniccola G.D., Castro M.G., Piovacari S.M.F., Horie L.M., Corrêa F.G., Barrere A.P.N., Toledo D.O. (2019). Current technologies in body composition assessment: Advantages and disadvantages. Nutrition.

[9] Coelho-Junior H.J., Picca A., Calvani R., Uchida M.C., Marzetti E. (2019). If my muscle could talk: Myokines as a biomarker of frailty. Exp. Gerontol..

[10] Xin C., Liu J., Zhang J., Zhu D., Wang H., Xiong L., Lee Y., Ye J., Lian K., Xu C. (2016). Irisin improves fatty acid oxidation and glucose utilization in type 2 diabetes by regulating the AMPK signaling pathway. Int. J. Obes..

[11] Kurdiova T., Balaz M., Vician M., Maderova D., Vlcek M., Valkovic L., Srbecky M., Imrich R., Kyselovicova O., Belan V. (2014). Effects of obesity; diabetes and exercise on Fndc5 gene expression and irisin release in human skeletal muscle and adipose tissue: In vivo and in vitro studies. J. Physiol..

[12] McPherron A.C., Lawler A.M., Lee S.J. (1997). Regulation of skeletal muscle mass in mice by a new TGF-beta superfamily member. Nature.

[13] Trendelenburg A.U., Meyer A., Rohner D., Boyle J., Hatakeyama S., Glass D.J. (2009). Myostatin reduces Akt/TORC1/p70S6K signaling; inhibiting myoblast differentiation and myotube size. Am. J. Physiol. Cell Physiol..

[14] Zimmers T.A., Davies M.V., Koniaris L.G., Haynes P., Esquela A.F., Tomkinson K.N., McPherron A.C., Wolfman N.M., Lee S.J. (2002). Induction of cachexia in mice by systemically administered myostatin. Science.

[15] Marzetti E., Calvani R., Cesari M., Buford T.W., Lorenzi M., Behnke B.J., Leeuwenburgh C. (2013). Mitochondrial dysfunction and sarcopenia of aging: From signaling pathways to clinical trials. Int. J. Biochem. Cell Biol..

[16] Groneberg D.A., Kindermann B., Althammer M., Klapper M., Vormann J., Littarru G.P., Döring F. (2005). Coenzyme Q10 affects expression of genes involved in cell signalling; metabolism and transport in human CaCo-2 cells. Int. J. Biochem. Cell Biol..

[17] Del Pozo-Cruz J., Rodríguez-Bies E., Ballesteros-Simarro M., Navas-Enamorado I., Tung B.T., Navas P., López-Lluch G. (2014). Physical activity affects plasma coenzyme Q10 levels differently in young and old humans. Biogerontology.

[18] Del Pozo-Cruz J., Rodríguez-Bies E., Navas-Enamorado I., Del Pozo-Cruz B., Navas P., López-Lluch G. (2014). Relationship between functional capacity and body mass index with plasma coenzyme Q10 and oxidative damage in community-dwelling elderly-people. Exp. Gerontol..

[19] Fischer A., Onur S., Niklowitz P., Menke T., Laudes M., Rimbach G., Döring F. (2016). Coenzyme Q10 Status as a Determinant of Muscular Strength in Two Independent Cohorts. PLoS ONE.

[20] Hernández-Camacho J.D., Bernier M., López-Lluch G., Navas P. (2018). Coenzyme Q10 Supplementation in Aging and Disease. Front. Physiol..

[21] Baumgartner R.N., Koehler K.M., Romero L., Garry P.J. (1996). Serum albumin is associated with skeletal muscle in elderly men and women. Am. J. Clin. Nutr..

[22] Visser M., Kritchevsky S.B., Newman A.B., Goodpaster B.H., Tylavsky F.A., Nevitt M.C., Harris T.B. (2005). Lower serum albumin concentration and change in muscle mass: The Health; Aging and Body Composition Study. Am. J. Clin. Nutr..

[23] Swaminathan R., Ho C.S., Donnan S.P. (1988). Body composition and plasma creatine kinase activity. Ann. Clin. Biochem..

[24] Guralnik J.M., Simonsick E.M., Ferrucci L., Glynn R.J., Berkman L.F., Blazer D.G., Scherr P.A., Wallace R.B. (1994). A short physical performance battery assessing lower extremity function: Association with self-reported disability and prediction of mortality and nursing home admission. J. Gerontol..

[25] Woo J., Leung J., Morley J.E. (2014). Validating the SARC-F: A suitable community screening tool for sarcopenia?. J. Am. Med. Dir. Assoc..

[26] Littarru G.P., Mosca F., Fattorini D., Bompadre S. (2007). Method to Assay Coenzyme Q10 in Blood Plasma or Blood Serum. U.S. Patent.

[27] Molyneux S.L., Young J.M., Florkowski C.M., Lever M., George P.M. (2008). Coenzyme Q10: Is there a clinical role and a case for measurement?. Clin. Biochem. Rev..

[28] Chen H.C., Huang C.C., Lin T.J., Hsu M.C., Hsu Y.J. (2019). Ubiquinol Supplementation Alters Exercise Induced Fatigue by Increasing Lipid Utilization in Mice. Nutrients.

[29] Chang P.S., Yen C.H., Huang Y.Y., Chiu C.J., Lin P.T. (2020). Associations between Coenzyme Q10 Status; Oxidative Stress; and Muscle Strength and Endurance in Subjects with Osteoarthritis. Antioxidants.

[30] de la Bella-Garzón R., Fernández-Portero C., Alarcón D., Amián J.G., López-Lluch G. (2022). Levels of Plasma Coenzyme Q10 Are Associated with Physical Capacity and Cardiovascular Risk in the Elderly. Antioxidants.

[31] Mancinelli R., Checcaglini F., Coscia F., Gigliotti P., Fulle S., Fanò-Illic G. (2021). Biological Aspects of Selected Myokines in Skeletal Muscle: Focus on Aging. Int. J. Mol. Sci..

[32] Chang J.S., Kim T.H., Nguyen T.T., Park K.S., Kim N., Kong I.D. (2017). Circulating irisin levels as a predictive biomarker for sarcopenia: A cross-sectional community-based study. Geriatr. Gerontol. Int..

[33] Baird M.F., Graham S.M., Baker J.S., Bickerstaff G.F. (2012). Creatine-kinase- and exercise-related muscle damage implications for muscle performance and recovery. J. Nutr. Metab..

[34] Rosalki S.B. (1998). Low serum creatine kinase activity. Clin. Chem..

[35] Flahault A., Metzger M., Chassé J.F., Haymann J.P., Boffa J.J., Flamant M., Vrtovsnik F., Houillier P., Stengel B., Thervet E. (2016). NephroTest study group. Low Serum Creatine Kinase Level Predicts Mortality in Subjects with a Chronic Kidney Disease. PLoS ONE.

[36] Kurita N., Kamitani T., Wada O., Shintani A., Mizuno K. (2021). Disentangling Associations Between Serum Muscle Biomarkers and Sarcopenia in the Presence of Pain and Inflammation Among Subjects with Osteoarthritis: The SPSS-OK Study. J. Clin. Rheumatol..

[37] Schalk B.W., Deeg D.J., Penninx B.W., Bouter L.M., Visser M. (2005). Serum albumin and muscle strength: A longitudinal study in older men and women. J. Am. Geriatr. Soc..

[38] Snyder C.K., Lapidus J.A., Cawthon P.M., Dam T.T., Sakai L.Y., Marshall L.M., Osteoporotic Fractures in Men (MrOS) Research Group (2012). Serum albumin in relation to change in muscle mass; muscle strength; and muscle power in older men. J. Am. Geriatr. Soc..

[39] Uemura K., Doi T., Lee S., Shimada H. (2019). Sarcopenia and Low Serum Albumin Level Synergistically Increase the Risk of Incident Disability in Older Adults. J. Am. Med. Dir. Assoc..

[40] Pravst I., Zmitek K., Zmitek J. (2010). Coenzyme Q10 contents in foods and fortification strategies. Crit. Rev. Food Sci. Nutr..

